# Temperature Compensation in Determining of Remazol Black B Concentrations Using Plastic Optical Fiber Based Sensor

**DOI:** 10.3390/s140915836

**Published:** 2014-08-27

**Authors:** Su Sin Chong, A.R. Abdul Aziz, Sulaiman W. Harun, Hamzah Arof

**Affiliations:** 1 Department of Chemical Engineering, Faculty of Engineering, University of Malaya, 50603 Kuala Lumpur, Malaysia; E-Mail: susin_2117@yahoo.com; 2 Department of Electrical Engineering, Faculty of Engineering, University of Malaya, 50603 Kuala Lumpur, Malaysia; E-Mails: swharun@um.edu.my (S.W.H.); _ahamzah@um.edu.my (H.A.)

**Keywords:** plastic optical fiber, environmental sensing, color sensor, tapered fiber, temperature compensation

## Abstract

In this study, the construction and test of tapered plastic optical fiber (POF) sensors, based on an intensity modulation approach are described. Tapered fiber sensors with different diameters of 0.65 mm, 0.45 mm, and 0.35 mm, were used to measure various concentrations of Remazol black B (RBB) dye aqueous solutions at room temperature. The concentrations of the RBB solutions were varied from 0 ppm to 70 ppm. In addition, the effect of varying the temperature of the RBB solution was also investigated. In this case, the output of the sensor was measured at four different temperatures of 27 °C, 30 °C, 35 °C, and 40 °C, while its concentration was fixed at 50 ppm and 100 ppm. The experimental results show that the tapered POF with *d* = 0.45 mm achieves the best performance with a reasonably good sensitivity of 61 × 10^−4^ and a linearity of more than 99%. It also maintains a sufficient and stable signal when heat was applied to the solution with a linearity of more than 97%. Since the transmitted intensity is dependent on both the concentration and temperature of the analyte, multiple linear regression analysis was performed to combine the two independent variables into a single equation. The resulting equation was then validated experimentally and the best agreement between the calculated and experimental results was achieved by the sensor with *d* = 0.45 mm, where the minimum discrepancy is less than 5%. The authors conclude that POF-based sensors are suitable for RBB dye concentration sensing and, with refinement in fabrication, better results could be achieved. Their low fabrication cost, simple configuration, accuracy, and high sensitivity would attract many potential applications in chemical and biological sensing.

## 1. Introduction

A large portion of reactive dyes is wasted during the dyeing process in textile manufacturing and up to 0.6–0.8 g dye/dm^3^ can be detected in the effluent [1]. As dyes cannot be easily removed from wastewater, they will cause environmental pollution due to their toxic and carcinogenic characteristics [2]. Stricter limits on wastewater discharges have resulted in a greater emphasis on wastewater monitoring and the treatment of effluent quality from the textile industry. However, the existing color-monitoring techniques, which involve scheduled sampling and chemical analysis, are costly and time consuming [3]. Therefore, a less cumbersome alternative is necessary and urgently needed to save cost, time, and manpower [4].

Tapered fiber optical sensors have been used in many physical, biological, and chemical sensing applications. They have high sensitivity, fast response, immunity to electromagnetic interference, and compact size [5]. As light propagates along the tapered section of the fiber, the evanescent field will leak from the core into the surrounding. The leaked wave optically interacts with surfactant molecules near the fiber surface, while propagating through the sensing region along the fiber with repeating reflections [5]. The portion of light that leaks travels and interacts with the ambience outside the fiber and is called the evanescent wave [6]. This finding enables tapered fiber to be used in many optic sensing applications.

Most fibers used for telecommunication are made of silica. However, tapered silica optical fiber (SOF) is fragile and easily disturbed by mechanical perturbation. To overcome this limitation, tapered plastic optical fibers (POF) are used to construct the sensors. Generally, POF are less expensive, have higher mechanical strength, and mass-produced components. Therefore, POF-based technology is very attractive for sensing deployment [7,8]. Furthermore, POF exhibit excellent corrode resistance when placed in direct contact with the sensing medium. The interaction between the evanescent wave of POF and the surrounding can be used in different ways to achieve a distributed sensing effect. This discovery has revolutionized the use of tapered POF without coating for environmental sensing as this method offers a number of advantages over the conventional methods that involve scheduled sampling and use of cuvettes.

When the sample has a distinctive absorption spectrum, changes in the spectrum can be detected by tapered POF through the transmitted signal. The sensitivity and level of interaction between the POF sensors and the surrounding environment also rely on the physical configuration of the sensors [9]. A number of applications of tapered plastics optical fiber (POF) sensors have been reported, such as for salinity detection [5,10], refractive index measurement [11], liquid level monitoring, humidity level assessment [12], and remote flood monitoring [13].

The objective of this study is to demonstrate the feasibility of using a tapered POF-based sensor to detect the concentration of RBB dye in aqueous medium. In order to obtain a more accurate model, the effect of the temperature of the medium on the output intensity is also investigated. Three linear equations are established to relate the output intensity to the concentration of the RBB solution and its temperature for three POF sensors with tapered waist diameters of 0.65 mm, 0.45 mm, and 0.35 mm. First, the concentration of the Remazol black B (RBB) was varied from 0 ppm to and 70 ppm by 10 ppm increments at ambient temperature. Then, the concentration of the Remazol black B (RBB) was fixed at 50 ppm and 100 ppm while the temperature was increased from 30 °C to 45 °C by 5 °C increment. Details of the experimental procedure and results are furnished in the following sections.

## 2. Experimental Design

Evanescent field sensing is particularly suitable for the measurement of chemical pollutants, when it is possible to treat the medium as a passive cladding [14]. Designing an effective evanescent field based sensor requires knowledge of certain parameters and how they influence the sensitivity and dynamic range of the sensor. When a tapered POF is immersed in analyte, the medium will act as a passive cladding. This will affect the amount of power loss from the signal that passes through the tapered region. The proposed sensor operates on the principle of intensity modulation, where the change in output intensity is investigated at various RBB concentrations. As the diameter of the tapered section is reduced, the sensitivity of the proposed sensor is investigated. In addition, the effect of the temperature of the analyte to the performance of the sensors is also analyzed.

### Multiple Linear Regression Analysis with Matrix Approach

Multiple linear regression (MLR) analysis involves exploring the linear relationship between a dependent variable and two or more independent variables. A general MLR model is usually written as:

(1) $$\begin{array}{cc} y_{i}=\beta_{0}+\beta_{1}X_{i1}+\cdots +\beta_{n}X_{in}+\epsilon_{i}, & i=1,2,\ldots ,k \end{array}$$

where *y_i_* is the dependent variable, *X_i_* are the independent variables, *β*_0_ is a constant, *β*_1_ and *β_n_* are coefficients of the independent variables and *∈_i_* is the error term [15]. In our study, the MLR analysis was performed using MATLAB (version 7.11.0, R2010b) software where the dependent variable is the normalized output intensity and the independent variables are the dye concentration and temperature. In matrix form, the equation can be written as:

(2) $$\begin{array}{cc} \left[\begin{array}{c} y_{1}\\ \vdots \\ y_{i} \end{array}\right]=\left[\begin{array}{c} \beta_{1}\\ \vdots \\ \beta_{n} \end{array}\right]\;\left[\begin{array}{ccc} 1 & C_{11} & T_{12}\\ \vdots & \vdots & \vdots \\ 1 & C_{i1} & T_{i2} \end{array}\right]+\left[\begin{array}{c} \epsilon_{1}\\ \vdots \\ \epsilon_{i} \end{array}\right] & i=1,2,\ldots ,k \end{array}$$

where *y*, *β*, *C*, *T*, and *ϵ* are dependent variable, coefficient of independent variables, RBB concentration variation, operational temperature variation and random errors of model, respectively.

## 3. Experiments

### 3.1. Preparation of Sensor Probe

**Figure 1.**
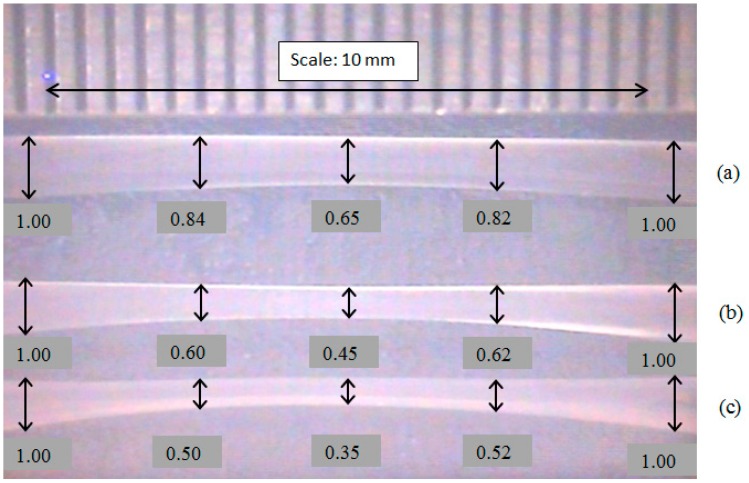
Three tapered POF based sensors with waist diameter (**a**) 0.65 mm (**b**) 0.45 mm and (**c**) 0.35 mm. All of the measurements done in these three tapered POF are in the unit of millimeters.

The POF used in this study was obtained from Edmund Optics (Model 02-534, USA) and it has an overall diameter of 1 mm, with refractive indices of 1.492 and 1.402 for the core and cladding, respectively. It was tapered using acetone, de-ionized water, and sand paper (Gold Cattle, 800 grit size) in accordance with chemical etching techniques [16,17]. First, a cotton bud was dipped in the acetone and used to wipe the surface of the POF. The acetone reacted with the outer surface of POF cladding to form a whitish layer. The POF was inspected under a microscope to ensure that the entire turned white surface of the target area had been polished by sand paper. This process was repeated until the desired diameter was obtained. Finally, total length of the tapered section (approximately 10 mm) was neutralized and cleaned by using deionized water. The tapered region and waist diameter of the POF were measured using a micrometer (Model 103-137, Mitutoyo, Japan) with a measuring range of 0.00–25.00 mm. The finished taper appears symmetrical enough under microscope (Model CT-2200, CT BRAND, China) inspection. Of course, a better way is to use the flame-brushing technique, which is more costly and complicated. Three tapered POFs with waist diameters of 0.65 mm, 0.45 mm, and 0.35 mm were prepared. The axial profile of a tapered fiber is shown in Figure 1, wherein a ruler with a scale of 0.5 mm was inserted for comparison purpose.

### 3.2. Preparation of Sensing Medium

Remazol Black B (RBB) was chosen because it is one of the most common dyes that are widely used in industries. It was supplied by Sigma-Aldrich and used without further purification. The stock solution was prepared by dissolving 0.5 g of RBB dye in one liter of distilled water and diluted to the desired concentrations, ranging between 0 ppm (distill water) and 70 ppm with 10 ppm increment.

### 3.3. Experimental Setup

**Figure 2.**
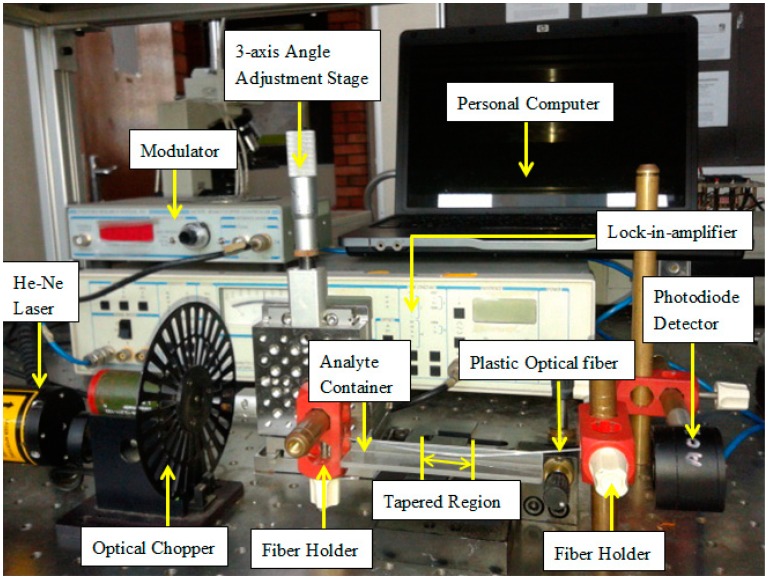
The tapered POF based sensor system for RBB concentration measurement.

The experimental setup of the sensing system that detects the concentration of the RBB solutions, from 0 ppm to 70 ppm, is depicted in Figure 2. The He-Ne laser light source (HRP050, Thorlabs, USA) operates at a wavelength of 633 nm, with an average output power of 5.5 mW. An optical chopper is stably rotating to modulate the intensity of the light beam and filter the harmonics from the power line. The He-Ne light source is launched into the tapered POF placed in a container filled with RBB solution. The output light is sent into the highly sensitive silicon photodiode detector (883 SL-OD3, Newport, UK) to be converted into an electrical signal. Then, the electrical signal, together with the reference signal from the optical chopper, is sent to the lock-in amplifier (SR-510, Stanford Research System, USA), which is used to improve the signal-to-noise ratio. Finally, the output signal from the lock-in amplifier is analyzed. The reference signal from the chopper is matched with the input electrical signal from the photodiode detector. The coupling of these two signals will remove the noise generated by the laser source, photo-detector, and the electrical amplifier in the photo-detector. Eventually, the output signal from the lock-in-amplifier is analyzed with a personal computer. To further increase the accuracy of the measurement, the light source, the POF on the fiber holder and the detector are positioned in a straight line to minimize bending loss that may occur in the POF.

## 4. Results and Discussion

**Figure 3.**
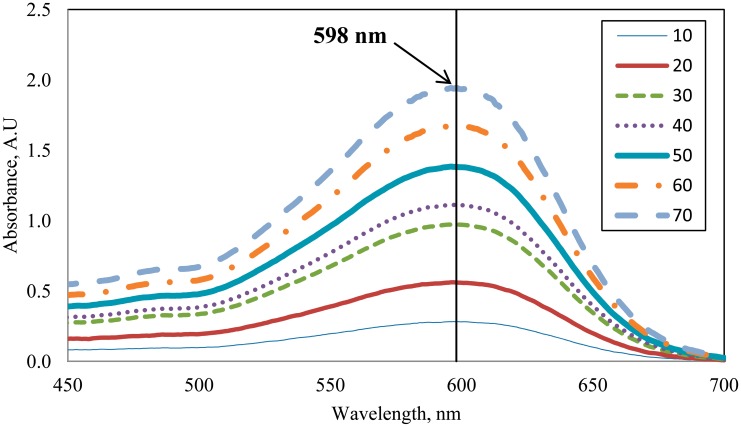
Absorption spectra of the RBB aqueous solution with concentrations ranging from 10 ppm to 70 ppm.

The RBB dye solution was chosen as a test medium as it has a distinctive absorption spectrum, which depends on the concentration. The absorption spectrum of the RBB sample was investigated using a UV-Vis spectrophotometer (Model SQ Pharo 300, Merck, Germany). Figure 3 shows the measured absorption spectrum of the RBB samples at various concentrations in ppm. As shown in the figure, RBB has a distinctive absorption spectrum ranging from 550 nm to 650 nm, where the peak absorption is at around 598 nm. Therefore, laser of 633 nm was used as the light source in this study since it falls within the absorption region of the sample.

### 4.1. Effect of Dye Concentration on Sensing

Charge interaction between the surface of the POF sensor and the molecules in the medium plays an important role that dictates the output intensity (rise linearly or decrease linearly) as a function of the analyte concentration [18]. Increasing the analyte concentration increases chromophore content of the solution. As a result, these positively polar charged chromophores will accumulate around the negatively polar charged surface of the fiber [19]. When the concentration of the analyte increases, the chromophores form a layer of electrostatic sheath around the surface of the fiber [20,21]. This might enlarge the pathway for the signal to pass through. Consequently, the output power also begins to rise linearly for this study, with increasingly concentrated sample solutions since the propagating light is more confined in the core of the tapered POF [19].

**Figure 4.**
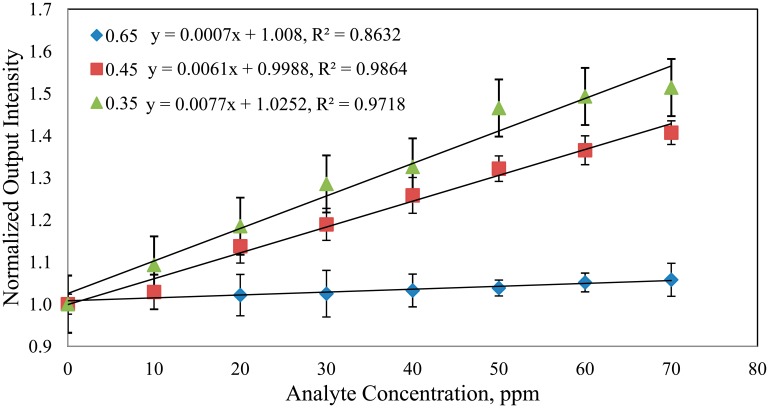
The variation of the normalized output intensity with tapered fiber diameters *d* = 0.65 mm, *d* = 0.45 mm and *d* = 0.35 mm for RBB at various concentrations.

The diameter of the POF influences the amounts of the evanescent field produced which in turn dictates the sensitivity of the sensor [18]. As seen in Figure 4, as the diameter of the tapered POF decreases, sensitivity increases. Sensors with *d* = 0.65 mm, *d* = 0.45 mm, *d* = 0.35 mm show relative sensitivity of 7.0 × 10^−4^, 61.0 × 10^−4^ and 77.0 × 10^−4^ respectively. In addition, a least square analysis was performed by assuming a square root dependence of output intensity on concentration. The tapered fiber with *d* = 0.65 mm, *d* = 0.45 mm and *d* = 0.35 mm shows linearity of 92.9%, 99.2% and 98.5%, respectively. The authors may conclude that the one with waist diameter of *d* = 0.65 mm does not demonstrate substantial sensitivity to the concentration of the analyte. As sensitivity increases as the fraction of the evanescent wave energy gets larger [8], the lack of sensitivity demonstrated by the sensors with thicker cladding is most probably due to lesser interaction between the evanescent field and the external medium [22].

### 4.2. Effect of Temperature on Dye Sensing

**Figure 5.**
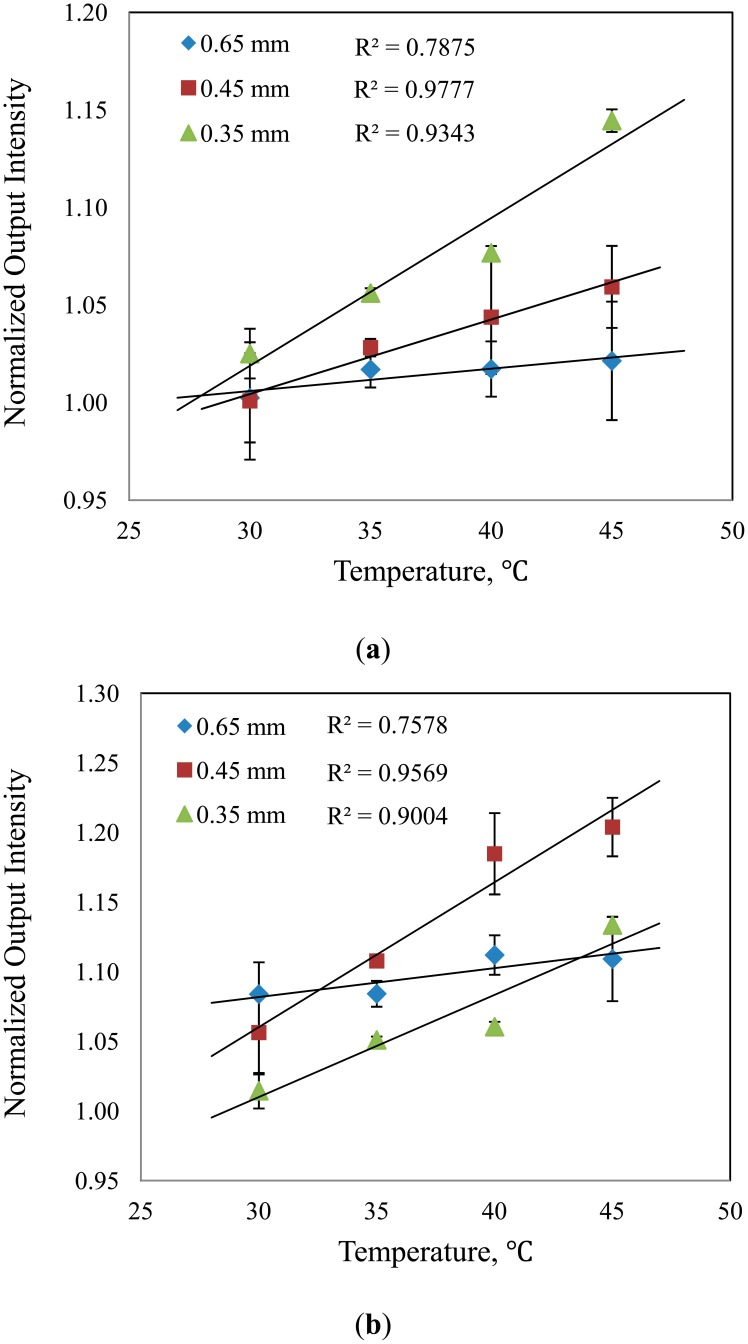
Responses of sensors toward temperature increment at (**a**) 50 ppm and (**b**) 100 ppm analyte.

One of the factors that affect the performance of the sensor and the behavior of the sensing medium is temperature. If the temperature of the analyte is known to vary considerably, corrective measure against temperature bias must be considered. Temperature change may affect the equilibrium of surrounding compound and thermal expansion coefficient of the tapered fiber [23]. To investigate the effect of temperature on RBB concentration sensing, an experiment was carried out where the RBB concentration was fixed at 50 ppm and 100 ppm, while the temperature was increased from 30 °C to 45 °C in 5 °C step. The results of the experiment are shown in Figure 5 where the normalized output intensity is shown to linearly increase with temperature. From the results, the sensor with *d* = 0.45 mm demonstrates the highest linearity compared to the others.

### 4.3. Model Validation

Since the transmitted intensity is linearly dependent on both the concentration and temperature of the RBB solution, an MLR analysis was performed to combine them in a single equation. The resulting equation (Equation (2)) contains a number of parameters that must be calculated from the experimental data. By writing the MATLAB script using matrix commands, the *β* coefficients of the independent variables can be found and the model for sensors with *d* = 0.65 mm, 0.45 mm, and 0.35 mm are given by Equation (3), Equation (4), and Equation (5). One additional step is to compute the maximum error, *∈* which indicates the accuracy of the model. The corresponding *∈* of Equations (3)–(5), are 0.04, 0.20, and 0.24, respectively. The resulting equation was then validated experimentally.

(3) $$y_{0.65\;mm}=0.9989-(0.0004\times \text{Temperature})+(0.0011\times \text{Concentration})$$

(4) $$y_{0.45\;mm}=1.3929-(0.0102\times \text{Temperature})+(0.0017\times \text{Concentration})$$

(5) $$y_{0.35\;mm}=1.6211-(0.0135\times \text{Temperature})+(0.00036\times \text{Concentration})$$

The validation experiment was conducted at 27 °C with RBB concentrations of 80 ppm, 90 ppm, and 100 ppm to verify the accuracy of the developed model. By utilizing Equation (6), the relative error (RE) between the calculated and experimental responses is calculated and shown in Table 1. From the results, the sensors with diameter of 0.65 mm, 0.45 mm, and 0.35 mm register discrepancies of less than 7%, 5%, and 6%, respectively. The best agreement between the calculated and experimental results was achieved by the sensor with *d* = 0.45 mm where the minimum discrepancy is less than 5%.

(6) $$\text{Relative error},\Delta =\frac{\left|I_{\text{experimental}}-I_{\text{calculated}}\right|}{I_{\text{calculated}}}x100\%$$

It is observed that the sensor with *d* = 0.45 mm shows the highest square of R, (R^2^ equal to 0.9864) and least square (R > 99%) values. Besides, the results obtained while operating in certain temperature range verify that the sensor in this diameter is adequately stable (R^2^ more than 0.95 for both concentrations of RBB) with a high repeatability. Furthermore, the relative error between the calculated and experimental values in validation dataset is the least compared to that of the other two diameters (RE less than 5%).

## 5. Conclusions

The performance of POF-based sensors of different diameters in measuring the concentration of RBB aqueous solution was investigated. The sensors are simple to fabricate and easy to handle with a good sensitivity, linearity and reproducibility. Although the sensitivity of the sensor is enhanced when the thickness of its cladding decreases, it becomes more vulnerable to the influence of the temperature of the analyte. In making appropriate adjustment to compensate for the temperature bias in the output readings, three model equations are introduced and validated. The POF with *d* = 0.45 mm displays good sensitivity and adequate stability within the tested temperature range. In addition, a good agreement between the calculated and measured responses was achieved with an average deviation of less than 5%. Knowledge of the optimum diameter of the tapered sensor for the operating temperature and RBB concentration range is useful for the development of an efficient sensor for the target application.

## Figures and Tables

**Table 1.** Model validation of the developed sensors.

**Concentration (ppm)**	**Temperature(**°C)	**Diameter (mm)**								

		0.65			0.45			0.35		

		Calculated ^(1)^	Experimental	Δ (%) ^(4)^	Calculated ^(2)^	Experimental	Δ (%) ^(4)^	Calculated ^(3)^	Experimental	Δ (%) ^(4)^
80	27	1.1125	1.0735	−3.51	1.4537	1.4158	−2.61	1.5257	1.4382	−5.73
90	27	1.1235	1.0568	−5.94	1.4707	1.4396	−2.11	1.5293	1.4734	−3.66
100	27	1.1345	1.0645	−6.18	1.4877	1.4585	−1.96	1.5329	1.6001	4.41

(1) Calculated according Equation (3);

(2) Calculated according Equation (4);

(3) Calculated according Equation (5);

(4) Relative error were calculated according Equation (6).

## Nomenclature

SOF: Silica optical fiber
POF: plastics optical fiber
RBB: Remazol black B
MLR: Multiple linear regression
He-Ne: Helium Neon
UV-Vis: Ultraviolet-Visible
*d*: Diameter of tapered fiber
ppm: Part per million or milligram per liter
R^2^: Square of R
R: Least square
RE: Relative error
